# A network pharmacology and molecular docking approach in the exploratory investigation of the biological mechanisms of lagundi (*Vitex negundo* L.) compounds against COVID-19

**DOI:** 10.5808/gi.22060

**Published:** 2023-03-31

**Authors:** Robertson G. Rivera, Patrick Junard S. Regidor, Edwin C. Ruamero Jr, Eric John V. Allanigue, Melanie V. Salinas

**Affiliations:** 1 Pharmaceutical Chemistry Department, College of Pharmacy, University of the Philippines Manila, Manila, Philippines; 2 Department of Pharmacology and Toxicology, College of Medicine, University of the Philippines Manila, Manila, Philippines; 3 Safety and Medical Affairs Department, Clinchoice Inc., Fort Washington, PA 19034, USA

**Keywords:** COVID-19, lagundi, molecular docking, network pharmacology, *Vitex negundo*

## Abstract

Coronavirus disease 2019 (COVID-19) is an inflammatory and infectious disease caused by severe acute respiratory syndrome coronavirus 2 virus with a complex pathophysiology. While COVID-19 vaccines and boosters are available, treatment of the disease is primarily supportive and symptomatic. Several research have suggested the potential of herbal medicines as an adjunctive treatment for the disease. A popular herbal medicine approved in the Philippines for the treatment of acute respiratory disease is *Vitex negundo* L. In fact, the Department of Science and Technology of the Philippines has funded a clinical trial to establish its potential as an adjunctive treatment for COVID-19. Here, we utilized network pharmacology and molecular docking in determining pivotal targets of *Vitex negundo* compounds against COVID-19. The results showed that significant targets of *Vitex negundo* compounds in COVID-19 are CSB, SERPINE1, and PLG which code for cathepsin B, plasminogen activator inhibitor-1, and plasminogen, respectively. Molecular docking revealed that α-terpinyl acetate and geranyl acetate have good binding affinity in cathepsin B; 6,7,4-trimethoxyflavanone, 5,6,7,8,3',4',5'-heptamethoxyflavone, artemetin, demethylnobiletin, gardenin A, geranyl acetate in plasminogen; and 7,8,4-trimethoxyflavanone in plasminogen activator inhibitor-1. While the results are promising, these are bound to the limitations of computational methods and further experimentation are needed to completely establish the molecular mechanisms of *Vitex negundo* against COVID-19.

## Introduction

Coronavirus disease 2019 (COVID-19) is a respiratory disease caused by the severe acute respiratory syndrome coronavirus 2 (SARS-CoV-2) virus and is highly infectious. Its symptoms are common to the common cold and may or may not require hospitalizations depending on the severity. Underlying medical conditions such as cardiovascular diseases, metabolic disorders, chronic respiratory diseases, and old age all contribute to the risk of developing a serious COVID-19 disease. Moreover, vaccination state can also affect how the person will respond to the disease, with vaccinated individuals having significantly less risk of being hospitalized or dying from the disease [1]. Up to date, there are no medications which can cure COVID-19 and the clinical care is mostly supportive in nature and are done to alleviate symptoms [2]. Several research have pointed out the possible utilization of herbal medicines to help in the management of COVID-19 [3,4]. One of which is *Vitex negundo* L. (VN) commonly known as lagundi in the Philippines. It is an herbal medicine approved for the treatment of mild to moderate cough and has been shown to elicit bronchodilating effects [5]. In the Philippines, there is an ongoing clinical trial for the potential of VN against COVID-19 [6]. However, up to date, the Philippine COVID-19 Living Clinical Practice Guidelines has not approved the use of VN as an adjunctive treatment for the disease because of the lack of evidence [7]. In fact, there are no published data about the effectiveness and molecular mechanisms of VN against COVID-19. To the best of our knowledge, only a few studies have explored the biological effects of VN compounds in COVID-19 which include the in silico screening of VN compounds against SARS-CoV-2 Mpro receptor [8] and against the papain-like protease of SARS-CoV-2 [9]. VN contains secondary metabolites primarily flavonoids and terpenoids. These groups of compounds have shown promising effects against reduction of exacerbation in COVID-19 primarily by inhibiting SARS-CoV-2 Mpro [10-12].

In line with the lack of data, the present work aims to give rationalize and give possible explanations on the mechanisms of the reported VN compounds against COVID-19 at the molecular level using network pharmacology and molecular docking.

## Methods

The methods presented here are iterations of previous work and were adapted from Zhang et al. [13] with some modifications.

### VN compounds identification, screening, and target determination

VN compounds were obtained from the Indian Medicinal Plants, Phytochemistry and Therapeutics database (IMPPAT | IMPPAT: Indian Medicinal Plants, Phytochemistry And Therapeutics (imsc.res.in)) [14,15]. Only secondary metabolites obtained from leaves and those obtained from unspecified parts of VN were considered. Duplicates were removed and the SMILES (Simplified Molecular Input Line Entry Specification) strings of the compounds were obtained from PubChem (https://pubchem.ncbi.nlm.nih.gov/) [16]. The SMILES strings were input in the Swiss ADME database (http://www.swissadme.ch/) [17] to screen compounds for oral bioavailability and drug likeness. Only compounds with good oral bioavailability [–0.7 < XLOGP3 (lipohilicity) < +5.0, 150 g/mol < MW < 500 g/mol, 20Å2 < TPSA (polarity) < 130Å2, –6 < LogS (ESOL) (insolubility) < 0, 0.25 < Fraction Csp3 (insaturation) < 1, 0 < number of rotatable bonds (flexibility) < 9] and those which passed the drug-likeness rules were considered for target identification. To determine the targets of the VN compounds, their SMILES strings were input in the Swiss Target Prediction database (http://www.swisstargetprediction.ch/) [18]. The file containing the targets were downloaded as CSV file and the targets with probability values greater than zero were collected and standardized as UniProt ID (https://www.uniprot.org/) [19].

### COVID-19 genes

Genes associated with COVID-19 were obtained from the MalaCards database (https://www.malacards.org/) [20] using COVID-19, severe acute respiratory syndrome, severe COVID-19, critical COVID-19, adult respiratory distress syndrome, non-severe COVID-19 and respiratory failure as keywords. The gene names were standardized as UniProt ID.

### VN targets in COVID-19

Using the FunRich application v.3.1.3 (http://www.funrich.org/) [21], a Venn diagram was constructed using the standardized gene names to determine the list of genes that are both targeted by VN compounds and are associated COVID-19. The targets were matched with the VN compounds and a compound-target network was constructed using Cytoscape v.3.9.1 (https://cytoscape.org/) [22]. Each node in the network represents VN compounds or their target gene whereas the edges are their interactions.

### Gene ontology and Kyoto Encyclopedia of Genes and Genomes pathways enrichment analysis

Gene ontology (GO) and Kyoto Encyclopedia of Genes and Genomes (KEGG) pathways enrichment analyses were conducted using the DAVID Bioinformatics Database (https://david.ncifcrf.gov/tools.jsp) [23,24]. The UniProt ID (identified as UniProt Accession by the database) of the COVID-19 targets of VN were input in the database and the GO analysis based on biological processes, cellular components, and molecular functions was conducted. Moreover, KEGG pathway enrichment analysis was also conducted to determine which pathways the targets are most likely to be involved in. The results of each analysis were ranked according to p-values (p ≤ 0.05) and visualized using R studio (https://www.rstudio.com/).

### Protein-protein interaction analysis

The UniProt ID of the VN targets in COVID-19 were input in the STRING database (https://string-db.org/) [25,26] using *Homo sapiens* as the filter organism, confidence score of 0.700, and false discovery rate stringency of 5%. The created protein-protein interaction network was extracted and visualized using Cytoscape. Each node represents the protein target whereas the edges represent their interactions. Using the Cytoscape Analyzer the centrality measures (degree centrality, betweenness centrality, and closeness centrality) were obtained and the targets were ranked according to these measures. The top three nodes for each centrality measure were obtained. Molecular Complex Detection (MCODE) plug-in in Cytoscape was also used to determine which nodes within the protein-protein interaction network are highly connected. The parameters were set as degree cut-off = 2, node score cut-off = 0.2, k-score = 2, and maximum depth = 100. Only one cluster resulted from the analysis.

### Molecular docking

#### Protein selection and preparation

After protein-protein interaction analysis, the genes were ranked based on the measures of centrality (degree, betweenness, closeness). Genes ranked with the highest measures of centrality were identified together with the proteins that they code. The 3-D crystal structures of the proteins were obtained from the Protein Data Bank (https://www.rcsb.org) [27]. The PDB file format of these proteins were downloaded and processed in Swiss PDB Viewer v4.1.0 [28] and Autodock Tools v1.5.6 [29]. The Swiss PDB Viewer was used to repair missing atoms in the structure. The standard protocol by Forli et al. (2016) [30] was followed using Autodock Tools. Briefly, the water molecules and heteroatoms were removed from the structure. Then, polar hydrogens were added, and the nonpolar hydrogens were merged. Gasteiger charges were then added, and the macromolecule was saved as PDBQT file.

#### Ligand preparation

Ligand structures were obtained from PubChem [16]. The 3D structures were downloaded as SDF files and were then converted into MOL2 files using OpenBabel v2.3.1 [31]. The structures were processed using Autodock Tools [29]. Following the protocol by Forli et al. (2016) [30], the nonpolar hydrogens were merged, polar hydrogens were added and the Kollmann charges were calculated. The structures were then saved as PDBQT file, and the energy minimized using MMFF94 forcefield.

#### Search space validation

For crystal structures of proteins with co-crystallized ligands, the ligands were redocked in the protein ensuring that the redocked pose has a root-mean-square deviation value of <2.0 Å. Redocking was done ten times to ensure that the results are consistent. As for the proteins 1qrz and 1huc which do not have co-crystallized ligands, CASTp3.0 webserver (http://sts.bioe.uic.edu/castp) was utilized to determine the ligand-binding pocket used for the docking procedure [32].

#### Docking procedure

All molecular docking procedure was conducted using Autodock Vina [33]. The default parameters were used, with the number of modes = 10, energy range = 3, exhaustiveness = 8. The xyz coordinates as well as the grid size (in Å) were adjusted to ensure that the ligands and that the amino acids reported to be involved in protein-ligand interactions fall within the set search space. Protein-ligand interactions were visualized using PyMol v.2.5.4 [34] and Discovery Studio Visualizer [35].

## Results

**Fig. 1.**
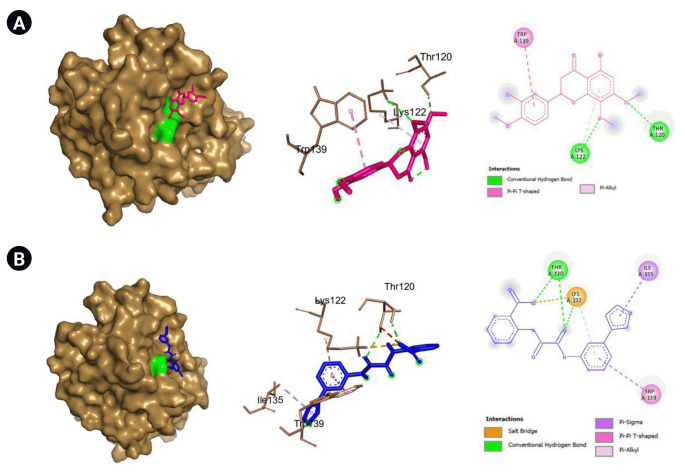
*Vitex negundo* compounds which have good oral bioavailability^a^ and passed the drug-likeness rules. ^a^Compounds with good oral bioavailability as indicated by Swiss ADME have the following medicinal chemistry properties: –0.7 < XLOGP3 < +5.0, 150 g/mol < MV < 500 g/mol, 20Å < TPSA < 130Å, –6 < ESOL < 0, 0.25 < fraction Csp3 < 1, and 0 < num. rotatable bonds < 9.

**Fig. 2.**
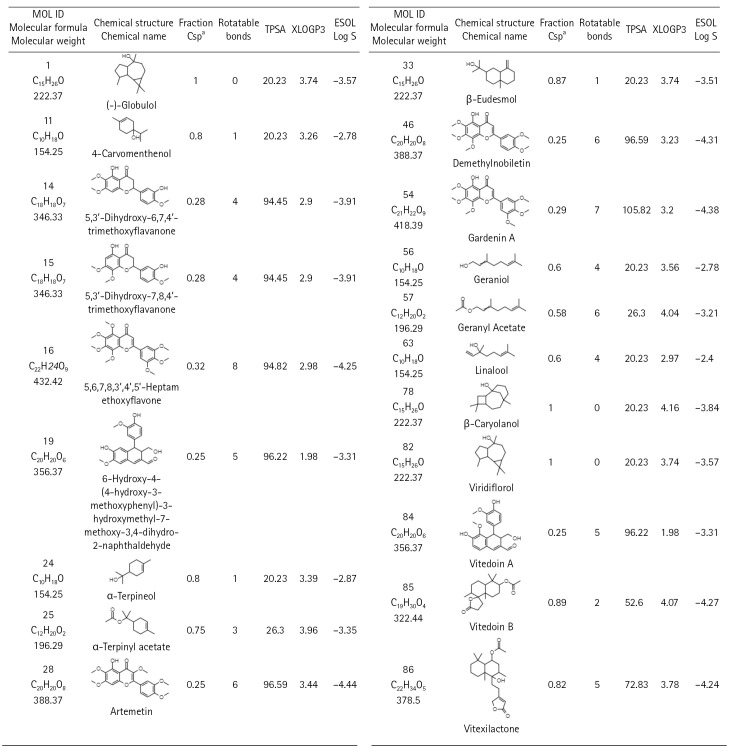
Venn diagram showing the intersection of the coronavirus disease 2019 (COVID-19) genes and the *Vitex negundo* L. compound targets.

**Fig. 3.**
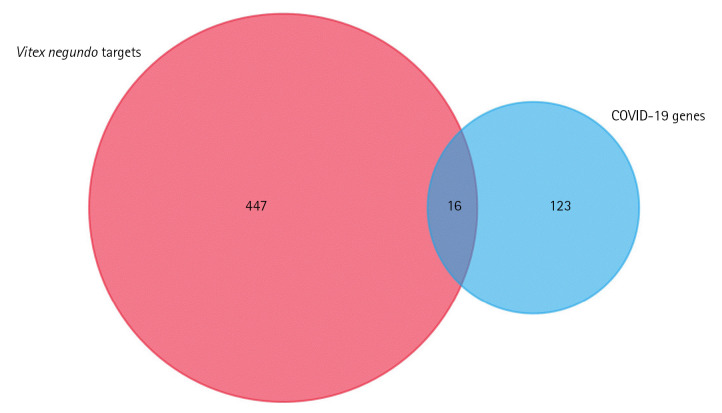
Compound target network of the reported *Vitex negundo* compounds and coronavirus disease 2019 targets. Represented in each node are the compounds (green) and the targets (pink). Each line represents the non-directional interaction between compounds.

There are 229 compounds of VN that are documented in the IMPPAT database and 89 of which are found either in leaves or in the unspecified parts of the plant. Shown in Fig. 1 are the 20 of these compounds which have good oral bioavailability and have passed the drug-likeness rules. All these compounds except vitedoin A have similar actives in the Swiss Target database with a total of 447 targets. In the MalaCards database, there are 123 genes related to COVID-19. Presented in Fig. 2 is the Venn diagram showing the overlap of the VN target genes and COVID-19–associated genes, with 16 genes that are targeted by VN compounds in COVID-19. The compound-target network indicates that there are multiple targets for the different VN compounds as shown in Fig. 3. The analysis of the compound-target network with each node representing either the target or the VN compound is shown in Table 1. Demethylnobiletin, α-terpinyl acetate, and F2 have the highest degree centrality; PLG, α-terpinyl acetate, and 5,3'-dihydroxy-6,7,4'-trimethoxyflavanone have the highest betweenness centrality; and SERPINE1, 5,3'-dihydroxy-7,8,4'-trimethoxyflavanone, and STAT1 have the highest closeness centrality. Supplementary Tables 1-4 contain the compounds from *Vitex negundo*, the genes associated with COVID-19, and the compound target network data.

**Fig. 4.**
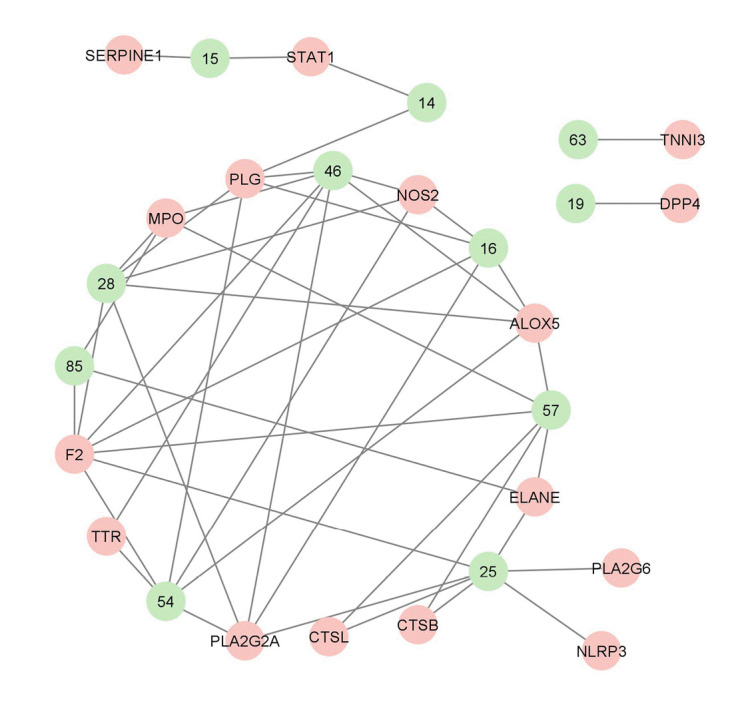
(A) Protein-protein interaction of the coronavirus disease 2019 targets of *Vitex negundo* compounds. The darker the color, the higher is the degree centrality. (B) One identified protein cluster in the protein-protein interaction network after the MCODE plug-in of Cytoscape was used.

The 16 genes that are targeted by VN compounds in COVID-19 were subjected to protein-protein interaction analysis and the results are shown in Table 2 and Fig. 4. The network analysis of protein-protein interaction showed that CTSB, SERPINE1, and PLG are the genes that have the highest degree, betweenness, and closeness centrality. This indicates that they are the pivotal proteins in COVID-19 that are targeted by VN compounds. In the said network, only one cluster can be seen which include PLA2G6, PLA2G2A, and ALOX5 after the MCODE cluster plug-in of Cytoscape was applied. These genes are highly enriched in the arachidonic acid metabolism. However, as presented in Table 2, their betweenness centrality values is zero.

**Fig. 5.**
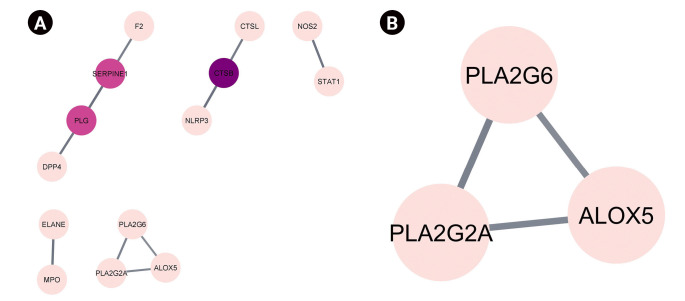
(A) Bubble plot of the biological processes (A), cellular components (B), molecular functions (C), highly associated with the coronavirus disease 2019 (COVID-19) proteins targeted by *Vitex negundo* compounds. (D) Bubble plot of the most enriched KEGG pathway associated with the COVID-19 proteins targeted by *Vitex negundo* compounds. Count is the number of differentially expressed genes (DEGs) enriched in the biological processes (A), cellular components (B), molecular functions (C), and pathways (D). The gene ratio indicates the ratio of enriched DEGs to background genes. The gene ratio indicates the ratio of enriched DEGs to background genes.

GO and KEGG pathway analyses were also conducted on these 16 genes. As shown in Fig. 5, these genes are highly associated with the biological processes involving proteolysis, negative regulation of fibrinolysis, and leukocyte migration involved in inflammatory response, among others. In cellular components, they are mostly associated with the extracellular region, extracellular space, extracellular exosome, etc. For molecular functions, they are associated with serine-type endopeptidase activity, protease binding, receptor binding, etc. KEGG enrichment analysis revealed that the most enriched pathways are the arachidonic acid metabolism and complement and coagulation pathways. Supplementary Tables 5–8 contain the tabular results of the enrichment analyses.

**Fig. 6.**
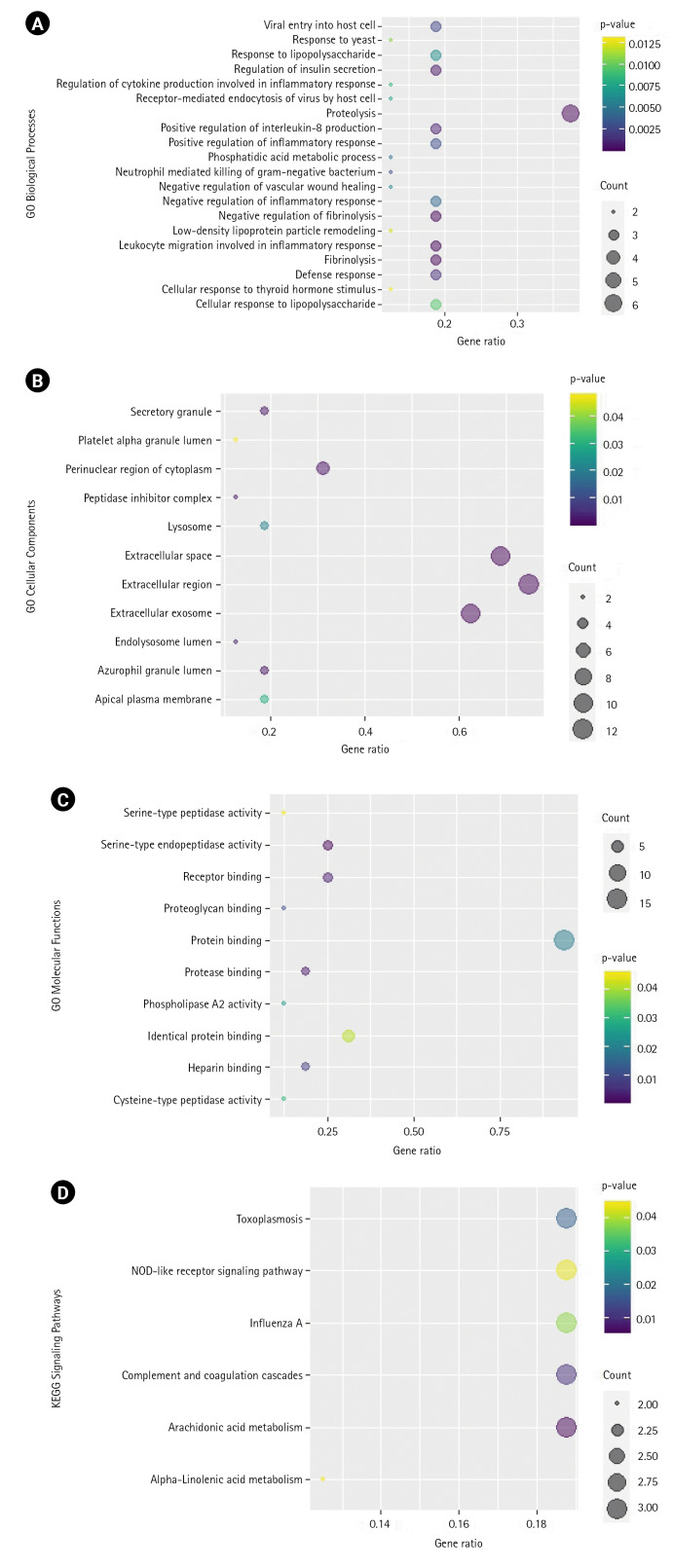
(A) Visualization of the molecular docking results of plasminogen (PDB ID:1qrz) and 6,7,4-trimethoxyflavanone. (B) Visualization of the molecular docking results of plasminogen (PDB ID:1qrz) and 5,6,7,8,3′,4′,5′-heptamethoxyflavone. (C) Visualization of the molecular docking results of plasminogen (PDB ID:1qrz) and artemetin. (D) Visualization of the molecular docking results of plasminogen (PDB ID:1qrz) and demethylnobiletin. (E) Visualization of the molecular docking results of plasminogen (PDB ID:1qrz) and gardenin A. (F) Visualization of the molecular docking results of plasminogen (PDB ID:1qrz) and geranyl acetate. Shown in the left is the 3-D representation of the docking, in the middle are the amino acid residues involved in the protein-ligand interaction and on the right are the intermolecular forces of attraction.

**Fig. 7.**
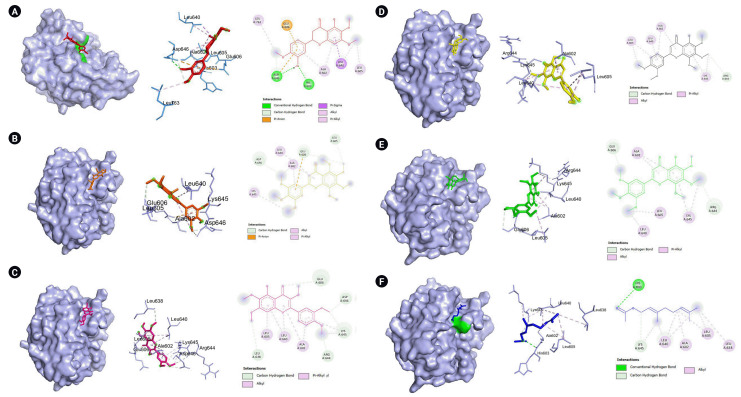
(A) Molecular docking result visualization of cathepsin B (PDB ID:1csb) and α-terpinyl acetate. (B) Molecular docking result visualization of cathepsin B (PDB ID:1csb) and geranyl acetate. (C) Molecular docking result visualization of cathepsin B (PDB ID:1csb) and the co-crystallized ligand EP048. Shown on the left is the 3-D representation of the docking of the ligand towards cathepsin B, in the middle are the amino acid residues involved in the protein ligand interactions, and on the right are the intermolecular forces of attraction.

**Fig. 8.**
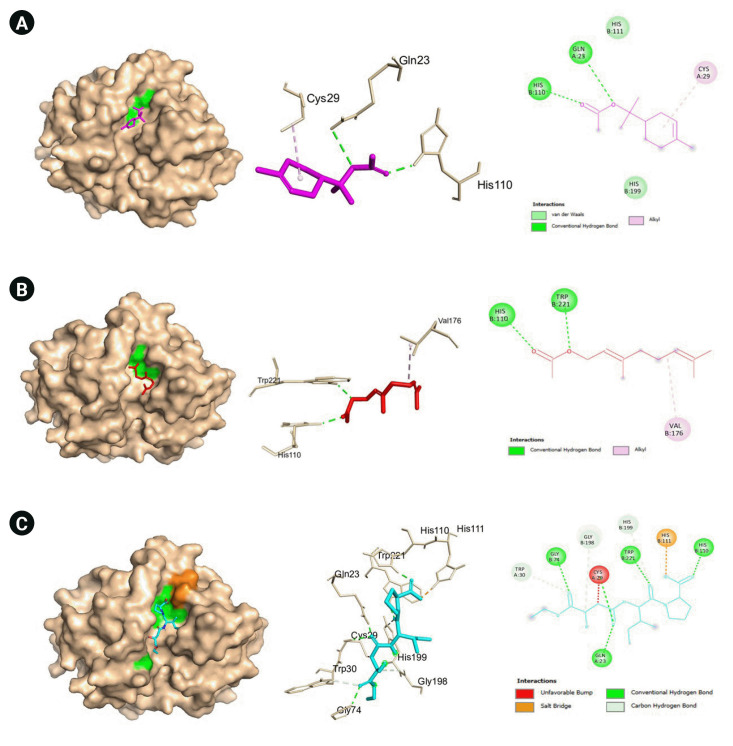
(A) Molecular docking result visualization of cathepsin B (PDB ID:1huc) and α-terpinyl acetate. (B) Molecular docking result visualization of cathepsin B (PDB ID:1huc) and geranyl acetate. Shown on the left is the 3-D representation of the docking of the ligand towards cathepsin B, in the middle are the amino acid residues involved in the protein ligand interactions, and on the right are the intermolecular forces of attraction.

**Fig. 9.**
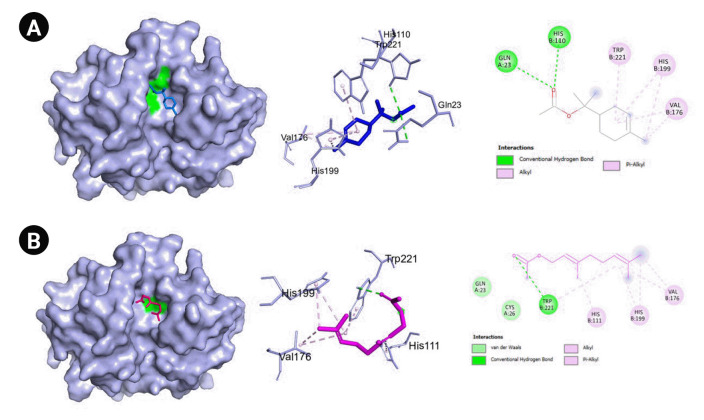
(A) Molecular docking result visualization of plasminogen activator inhibitor-1 (PDB ID:4aqh) and 7,8,4-trimethoxyflavanone. (B) Molecular docking result visualization of plasminogen activator inhibitor-1 (PDB ID:4aqh) and the co-crystallized ligand TB71. Shown on the left is the 3-D representation of the docking of the ligand towards cathepsin B, in the middle are the amino acid residues involved in the protein ligand interactions, and on the right are the intermolecular forces of attraction.

**Fig. 10.**
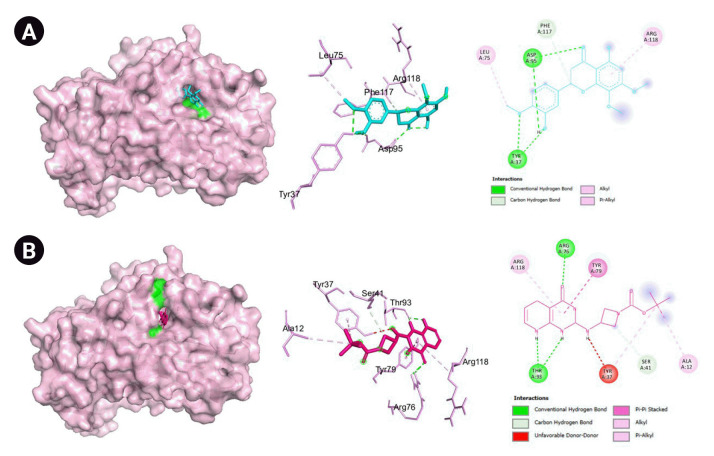
(A) Molecular docking result visualization of plasminogen activator inhibitor-1 (PDB ID:7aqf) 7,8,4-trimethoxyflavanone. (B) Molecular docking result visualization of plasminogen activator inhibitor-1 (PDB ID:7aqf) and its co-crystallized ligand RV2401. Shown on the left is the 3-D representation of the docking of the ligand towards cathepsin B, in the middle are the amino acid residues involved in the protien ligand interactions, and on the right are the intermolecular forces of attraction.

The genes with the highest centrality measures are CSB, *SERPINE1*, and PLG which codes for cathepsin B, plasminogen activator inhibitor-1, and plasminogen, respectively. The protein structures utilized were PDB ID: 1qrz (plasminogen), PDB ID: 1csb and 1huc (cathepsin B), and PDB ID: 4aqh and 7aqf (plasminogen activator inhibitor-1). For 1csb, the co-crystallized ligand is compound EP048; compound TB71 in 4aqh; and compound RV2401 in 7aqf. The results of the molecular docking are summarized in Table 3 and Figs. 6–10. As seen in Table 3, majority of the protein-ligand interactions involve hydrogen bonding and hydrophobic interactions. While an unfavorable interaction is observed between compound TB71 and 4aqh, the negative binding energy suggests that these were offset by the other favorable intermolecular forces of attraction. In each of the figures in Figs. 6–10, the three-dimensional surface structure of the proteins (left), the amino acid residues involved in the protein-ligand interactions (center) as well as the two-dimensional map of the interactions showing the intermolecular forces of attraction are presented.

## Discussion

COVID-19 is an inflammatory disease with a complicated pathophysiology. It has a systemic effect in the body and physiological functions. Hence, an integrative approach is needed to determine pivotal targets. Network pharmacology and molecular docking is an integrative approach in the determination of the pivotal targets of multiple compounds using a combination of bioinformatics and computational approach. In the Philippines, there is an ongoing clinical trial on the potential of VN against COVID-19 under the Department of Science and Technology. However, its molecular mechanism has not been elucidated yet. In the present work, network pharmacology and molecular docking were utilized to determine the pivotal proteins in COVID-19 that can be targeted by VN compounds. Network pharmacology combines computational methods, graph theory, and bioinformatics so that diseases are not viewed by one-target-one drug approach rather through a holistic approach [36]. In tandem with this approach, molecular docking, aims to assess the favorability of the binding of the small ligands through computational approach and algorithms. The calculated binding energy and poses as well as the protein-ligand interactions serve as the basis for the favorability of the binding. That is, the more negative the binding affinity is, the more favorable the ligand binds to the receptor [37]. As previously mentioned, three genes were identified to be important targets of VN compounds in COVID-19 which includes CTSB, SERPINE1, and PLG.

PLG codes for the protein plasminogen which is the precursor of plasmin, that in turn functions in clot dissolution and extracellular matrix protein degradation. Moreover, plasmin may activate intracellular signaling pathways which include expression of proinflammatory genes [38]. It was reported that plasminogen and plasmin contribute to susceptibility to COVID-19 and that in vitro experiments revealed that plasmin cleaves protein S of SARS-CoV allowing it to penetrate the cellular host [39]. However, the benefit or risk of plasminogen or plasmin (and the fibrinolytic system) to COVID-19 patients remain uncertain because of conflicting studies [40] and merit further investigation. For example, inhalation of plasminogen has been shown to improve oxygen levels in a small group of patients with moderate COVID-19 [41] and that low levels of plasminogen was reported to be correlated with mortality in COVID-19 patients [42]. In the present study, six VN compounds were shown to have favorable binding affinity towards plasminogen with calculated binding energies ranging from –5.4 to –6.3 kcal/mol as shown in Table 3. Moreover, as presented in Fig. 6, they interact with important amino acids such as Asp646 and His603 which are part of the catalytic triad [43]. Additionally, the amino acid residues that are present in the protein-ligand interactions between these VN compounds (6,7,4-trimethoxyflavanone, 5,6,7,8,3′,4′,5′-heptamethoxyflavanone, artemetin, demethylnobiletin, gardenin A, and geranyl acetate) and the plasminogen molecule includes the residues that are part of the 94-shunt (residues 641–645), and the 60-loop (residues 606–610) which are important in ligand binding [44]. However, further experimental investigations are needed to confirm their binding and activity towards plasminogen because up to date, no literature has been reported.

Another pivotal protein is cathepsin B which is a member of cysteine proteases that exhibits both endopeptdase and exopeptidase activity. It is localized to the lysosomal compartment and is involved primarily in cellular turnover of proteins both extracellularly and intracellularly. Moreover, it also serves its function in pro-hormone and proenzyme activation, antigen processing, and inflammatory processes. Cathepsin B has been implicated in diseases such as Alzheimer’s disease, some cancers including brain and breast cancers, pancreatitis, and arthritis [45]. In COVID-19, CTSB is upregulated. It also interacts with proteins that are involved in antigen processing, presentation, and inflammatory responses. GO analyses also revealed that CTSB is highly enriched in cytokine production regulation [46]. Moreover, CTSB is involved in the entry of the COVID-19 virus within cells via the endosomal pathway by cleaving and activating protein S. Additionally, expression of CTSB in COVID-19 may also lead to an increased risk of COVID-19 infection [47]. Hence, the inhibition of cathepsin B has a positive effect on the alleviation of COVID-19. In our current work, two VN compounds have been found to bind favorably to cathepsin B which include geranyl acetate and α-terpinyl acetate with a little over 5.0 kcal/mol binding energies as shown in Table 3. The protein-binding site of cathepsin B is composed of Gln23, Gly24, Gly27, Cys29, ASn72, Gly74, His110, Glu122, Met196, Gly198, His199, and Trp221. The amino acids Gln23, Gly74, Gly198, His110, His111, and Met196 are reported to be involved in hydrogen bonding with inhibitors. Moreover, most cathepsin B inhibitors interact with Cys29, Gln23, Gly198, His199, and Trp221 [48]. As seen in Table 3 and Figs. 7 and 8, most of these amino acids are also present in the protein-ligand interactions between geranyl acetate, α-terpinyl acetate, and cathepsin B. These suggest that both geranyl acetate and α-terpinyl acetate may be possible candidates as inhibitors for further experimental testing against cathepsin B in the context of COVID-19. To the best of our knowledge, there were no reports confirming the activity of geranyl acetate and α-terpinyl acetate against cathepsin B. The closest compound related to the terpenoids geranyl acetate and α-terpinyl acetate known to inhibit cathepsin B are ursolic acid [49] and frondoside A [50], both of which are triterpenes.

SERPINE1 codes for the protein plasminogen activator inhibitor 1 (PAI-1) which is a serine protease inhibitor that inhibits the activation of the tissue-type plasminogen activator, hence preventing the fibrinolytic process [51]. Abnormalities in the interplay between plasminogen activators and impairment in fibrinolysis have been seen in COVID-19 patients leading to thrombotic events and coagulopathies. The level of PAI-1 is increased in COVID-19 patients and is associated with worse respiratory status and poor clinical outcomes [52,53]. Moreover, increased levels of PAI-1 due to STAT3 activation may inhibit tissue-type plasminogen activator and urokinase-type plasminogen activator that eventually leads to thrombosis [54]. Hence, PAI-1 can be a potential target for decreasing the risk of thrombosis and coagulopathies brought about by COVID-19 complications. Here, 5,3'-dihydroxy-7,8,4-trimethoxyflavanone binds favorably to PAI-1 with a calculated binding affinity of –6.9 kcal/mol as shown in table. However, as presented in Table 3 and seen in Figs. 9 and 10, the amino acid residues involved in the protein-ligand interactions of inhibitors (TB701 and RV2401) and PAI-1 are not found in the protein-ligand interactions of the flavanone and the said receptor. Moreover, in a structure activity relationship of PAI-1 inhibitors conducted by Yamaoka et al. [55], the presence of thiophene rings with bulky or hydrophobic substituents and a carboxylic acid moiety make the compound a potent PAI-1 inhibitor. These structural features are not present in the flavanone structure as shown in Fig. 1. This may indicate that the flavanone binds in a different conformation than that of the inhibitors and may or may not exhibit an activity. In cell studies conducted by Gimenez-Bastida et al. [56], flavonoid-rich Citrus extracts were able to downregulate PAI-1 and matrix metallopeptidase 12 (MMP-12). Also, in another study, it was reported that baicalein, another flavonoid was able to inhibit PAI-1 in a dose-dependent manner [57]. However, there were no reports on the activity of 5,3’-dihydroxy-7,8,4-trimethoxyflavanone towards PAI-1 and this warrants further investigation and experimentation.

VN locally known as lagundi in the Philippines is approved for therapeutic use against cough brought about by the common colds, flu, and moderate bronchitis. Currently, its activity against COVID-19 is being studied by the Philippine Council for Health Research and Development under the Department of Science and Technology in the Philippines. However, its biological effects and mechanisms towards COVID-19 are yet to be elucidated. With the use of network pharmacology and molecular docking in the present work, it was found that the reported compounds of VN can possibly affect the molecular pathogenesis of COVID-19 by acting on plasminogen activator inhibitor-1, plasminogen, and cathepsin B. However, the limitation of this study relies on the fact that these are computational predictions and further experimentations are needed. Moreover, the quantity and potency of these compounds present in *Vitex negundo*, and the possibility of synergism further complicates the establishment of the potential of VN against COVID-19.

## Figures and Tables

**Table 1.** Network analysis of the compound-target network

Node	Degree	Betweenness centrality	Closeness centrality
α-Terpinyl acetate	7	0.27	0.39
Demethylnobiletin	7	0.16	0.46
F2	7	0.23	0.47
Artemetin	6	0.11	0.44
Gardenin A	6	0.12	0.44
Geranyl acetate	6	0.13	0.38
5,6,7,8,3′,4′,5′-Heptamethoxyflavone	5	0.08	0.42
ALOX5	5	0.06	0.40
PLA2G2A	5	0.12	0.43
PLG	5	0.32	0.40
MPO	4	0.05	0.39
NOS2	4	0.01	0.35
Vitedoin B	3	0.02	0.34
ELANE	3	0.02	0.32
5,3′-Dihydroxy-6,7,4'-trimethoxyflavanone	2	0.25	0.31
5,3'-Dihydroxy-7,8,4'-trimethoxyflavanone	2	0.09	0.21
CTSB	2	0.01	0.31
CTSL	2	0.01	0.31
STAT1	2	0.17	0.25
TTR	2	0.00	0.33
6-Hydroxy-4-(4-hydroxy-3-methoxyphenyl)-3-hydroxymethyl-7-methoxy-3,4-dihydro-2-naphthaldehyde	1	0.00	1.00
Linalool	1	0.00	1.00
DPP4	1	0.00	1.00
NLRP3	1	0.00	0.29
PLA2G6	1	0.00	0.29
SERPINE1	1	0.00	0.17
TNNI3	1	0.00	1.00

**Table 2.** Centrality measures of the different proteins in COVID-19 that are targeted by *Vitex negundo* compounds

Gene	Degree	Betweenness centrality	Closeness centrality
*CTSB*	2	1.00	1.00
*SERPINE1*	2	0.67	0.75
*PLG*	2	0.67	0.75
*NOS2*	1	0.00	1.00
*TNNI3*	0	0.00	0.00
*MPO*	1	0.00	1.00
*PLA2G2A*	2	0.00	1.00
*STAT1*	1	0.00	1.00
*CTSL*	1	0.00	0.67
*ALOX5*	2	0.00	1.00
*F2*	1	0.00	0.50
*TTR*	0	0.00	0.00
*ELANE*	1	0.00	1.00
*NLRP3*	1	0.00	0.67
*PLA2G6*	2	0.00	1.00
*DPP4*	1	0.00	0.50

COVID-19, coronavirus disease 2019.

**Table 3.** Calculated binding energies and the amino acid residues involved in the protein-ligand interactions between the *Vitex negundo* compounds and the pivotal proteins in COVID-19

Compound	Binding energy (kcal/mol)	Hydrogen bonding	Hydrophobic interactions	Others
PLG: Plasminogen (PDB ID: 1qrz)				
6,7,4-Trimethoxy-flavanone	–6.3	Asp646, His603	Ala602, Leu605, Leu640, Leu763	Glu606 (pi-anion)
5,6,7,8,3',4',5'-Hepta-methoxyflavone	–5.5	-	Ala602, Asp646, Glu606, Leu605, Leu640, Lys645	
Artemetin	–6.3	-	Ala602, Arg644, Asp646, Glu606, Leu605, Leu638, Leu640, Lys645	
Demethyl-nobiletin	–5.7	-	Ala602, Arg644, Leu605, Leu640, Lys645	
Gardenin A	–5.6	-	Ala602, Arg644, Glu606, Leu605, Leu640, Lys645	
Geranyl acetate	–5.4	His603	Ala602, Leu605, Leu638, Leu640, Lys645	
CTSB: Cathepsin B (PDB ID: 1csb)				
α-Terpinyl acetate	–5.5	Gln23, His110	Cys29	
Geranyl acetate	–5.3	His110, Trp221	Val176	
EP048^a^	–7.4	Gln23, Gly74,	Gly198, His199, Trp30	His111 (salt bridge)
		His110, Trp221		
CTSB: cathepsin B (PDB ID: 1huc)				
α-Terpinyl acetate	–5.5	Gln23, His110	His199, Trp221, Val176	
Geranyl acetate	–5.1	Trp21	His111, His199, Val176, Cys26, Gln23	
SERPINE1: Plasminogen activator inhibitor-1 (PDB ID: 4aqh)				
7,8,4-Trimethoxy-flavanone	–6.9	Asp95, Tyr37	Arg118, Leu75, Phe117	
TB71^a^	–8.9	Arg76, Thr93	Ala12, Arg118, Ser41, Tyr79	Tyr37 (unfavorable donor-donor)
SERPINE1: Plasminogen activator inhibitor-1 (PDB ID: 7aqf)				
7,8,4-Trimethoxy-flavanone	–6.5	Lys122, Thr120	Trp139	
RV2401^a^	–7.1	Lys122, Thr120	Ile135, Trp139	Lys122 (salt bridge)

COVID-19, coronavirus disease 2019.

a Co-crystallized ligands.

## Supplementary Materials

Supplementary Table 1. Vitex negundo compounds and their Swiss ADME results

Supplementary Table 2. Genes associated with Covid-19

Supplementary Table 3. Targets of Vitex negundo compounds

Supplementary Table 4. Data used in constructing the compound-target network

Supplementary Table 5. Gene ontology (biological processes) results

Supplementary Table 6. Gene ontology (cellular components) results

Supplementary Table 7. Gene ontology (molecular functions) results

Supplementary Table 8. KEGG pathway analysis results
